# Epidemiological characteristics and spatiotemporal patterns of hand, foot, and mouth disease in Hubei, China from 2009 to 2019

**DOI:** 10.1371/journal.pone.0287539

**Published:** 2023-06-23

**Authors:** Wuwei Wang, Mark W. Rosenberg, Hongying Chen, Shengsheng Gong, Mengmeng Yang, Dacai Deng

**Affiliations:** 1 Institute of China Rural Studies, Central China Normal University, Wuhan, Hubei, China; 2 Institute of Sustainable Development & Department of Geography, Central China Normal University, Wuhan, Hubei, China; 3 Department of Geography and Planning, Queen’s University, Kingston, Ontario, Canada; 4 Center for Disease Control and Prevention of Hubei Province, Wuhan, Hubei, China; Shanghai Public Health Clinical Center, Fudan University, CHINA

## Abstract

**Background:**

Hand, foot, and mouth disease (HFMD) is a public health issue in Hubei and studies of- spatiotemporal clustering at a fine scale are limited. The purpose of this research was to analyze the epidemiological characteristics, temporal variation characteristics, and spatiotemporal clustering of HFMD cases at the town level from 2009 to 2019 to improve public health outcomes.

**Methods:**

Mathematical statistics, a seasonal index, wavelet analysis, and spatiotemporal scans were used to analyze epidemiological characteristics, time series trends, and spatiotemporal clusters of HFMD in Hubei.

**Results:**

EV-A71 (Enterovirus A71) and CVA16 (Coxsackievirus A16) constitute the two primary pathogens of the HFMD epidemic in Hubei, among which EV-A71 is the dominant pathogen, especially in 2016. In terms of age distribution, a major peak occurred at 0–5 years and a very small increase appeared at 25–35 years, with the former having a higher incidence among males than females and the latter having the opposite difference between males and females. The number/rate of HFMD cases exhibited a considerable increase followed by a moderate decline from 2009 to 2019, with the first large peak in April-July and a smaller peak in November-December. HFMD in Hubei exhibited the characteristics of a 270-day cycle with multiscale nesting, which was similar to the periodicity of HFMD cases caused by EV-A71 (9 months). Cities with a higher incidence of HFMD formed a part of an “A-shaped urban skeleton”. Subdistricts had the highest incidence of HFMD, followed by towns and villages. The spatiotemporal scan results showed one most likely cluster and 22 secondary clusters, which was consistent with the geographic location of railways and rivers in Hubei.

**Conclusions:**

These findings may be helpful in the prevention and control of HFMD transmission and in implementing effective measures in Hubei Province.

## Introduction

Hand, foot, and mouth disease (HFMD) is an acute contagious viral disease mainly caused by human enterovirus 71 (EV-A71) and coxsackievirus A16 (CVA16) that infects children under 5 years of age [1]. The most common mechanisms of transmission include close human contact, infected objects, and fecal-oral transfer [2]. Infants and children are susceptible to this disease whose main symptoms are herpes or rash on the hands, feet, and mouth. Severe patients occasionally have complications such as myocarditis, pulmonary edema, and aseptic meningitis [3, 4]. HFMD was first documented in New Zealand in 1957 [5] and is now prevalent worldwide, especially in the Asia-Pacific region, such as Singapore [6], Thailand [7], mainland China [8, 9], Taiwan [10], Malaysia [11], and Japan [12]. HFMD was first reported in Shanghai in 1981 and then spread throughout China [13]. HFMD has become a major public health issue in China. Thirty four provincial administrative units have reported cases of HFMD, including Beijing [14], Tianjin [15], Hebei [16], Shandong [17], Hubei [18], and Guangdong [19]. The Chinese Ministry of Health declared HFMD a notifiable class C infectious disease in May 2008 [20]- following an outbreak of HFMD in Fuyang city that resulted in 6,049 cases between March 1 and May 9, 2008 [21].

HFMD poses a great threat to the health of children in Hubei Province. In recent years, the incidence rate of HFMD in Hubei has risen rapidly. HFMD ranked first in the obligatorily reported infectious disease list for several years, and the incidence rate of severe cases in Central China is higher than that in other areas [22]. From 2009 to 2015, Hubei reported a total of 496,096 cases of HFMD, increasing from 33,211 cases in 2009 to 77,245 cases in 2015, with a male-to-female ratio of 1.69:1, mainly among children aged 5 and below [23]. The number of HFMD cases in Hubei climbed to 120,470, with a 133.30/100,000 incidence rate in 2016 [24].

Previous papers about HFMD in Hubei have given more attention to etiological and epidemiological factors, the latter with a crude space-time scale such as a city scale [18, 25]. In contrast, there have been few studies on the spatial and temporal distribution of HFMD at the town and subdistricts scales (i.e., finer geographic scales), and regional differences in pathogenetic characteristics of HFMD remain poorly understood. Vaccination approaches for dealing with HFMD against EV-A71 were implemented in Hubei after July 2016. All children are eligible for vaccination if caregivers are willing to pay because the EV-A71 vaccine is identified as a class II (not free) vaccine. Studies of the epidemiological characteristics of HFMD in Hubei after 2016 are limited and need to be explored. Compared with previous studies, this study aimed to analyze epidemiological features, temporal characteristics using weeks as the time scale, and space-time clusters using towns as the spatial scale of HFMD in Hubei from 2009 to 2019. The results could be helpful for reducing the incidence rate of HFMD and facilitate prevention and control measures to avoid outbreaks in key areas and time periods in Hubei Province.

## Materials and methods

### Study area

Hubei Province is situated in the middle reaches of the Yangtze River and features a subtropical monsoon climate. There are 12 prefecture-level cities in the province (Wuhan, Huanggang, Ezhou, Huangshi, Xianning, Yichang, Xiangyang, Jingzhou, Jingmen, Suizhou, Xiaogan, Shiyan), one autonomous prefecture (Enshi), three municipalities (Tianmen, Qianjiang, Xiantao), one forested region (Shennongjia). The population of Hubei was 59.27 million at the end of 2019, with a 61% urbanization rate (Statistical Bulletin of National Economic and Social Development of Hubei Province 2019). Economic development in Hubei is uneven, with a gradient difference between the east, middle, and west, with a higher economic level in the central section area (the area between the three central cities of Wuhan, Xiangyang, and Yichang) and a lower economic level in the east and west areas.

### Data

The data were collected from the Hubei Provincial Center for Disease Control and Prevention (https://www.hbcdc.com/), and included a total of 878,257 HFMD cases from January 2009 to December 2019. The sex ratio of HFMD cases was also obtained from the above department. The cases of HFMD were aggregated data of provinces, cities, and counties and did not contain personal privacy information. The disease was diagnosed based on an epidemiological investigation of clinical symptoms, laboratory test results, and the Ministry of Health guidelines for the diagnosis and treatment of HFMD. The data of laboratory-confirmed cases from 2010 to 2019 were used to analyze the pathogenic characteristics of HFMD in Hubei, since the infectious disease reporting system had no data on laboratory-confirmed cases in 2009. The date of onset was used to determine the occurrence of cases. Demographic data for Hubei were obtained from the Hubei Province Township Statistical Yearbook.

### Statistical analysis

Descriptive statistical analysis was conducted using Origin 9.0 software (https://www.originlab.com/) to describe the characteristics of the reported HFMD cases in Hubei from 2009 to 2019. Graphs of the reported cases were drawn to show the sex and age differences in HFMD per year.

### Seasonal index

The seasonal index was used to examine the seasonal characteristics of the HFMD incidence rate, with the following formula: seasonal index = average incidence of the same month of each year/ monthly average incidence [26]. If the index is greater than 100%, the month is in its peak season of onset, and if it is less than 100%, the month is in its off-season. The seasonal index’s coefficient of variation also shows the seasonality of HFMD [23]. The stronger the seasonality is, the higher the coefficient of variation. The formula is as follows: coefficient of variation = (standard deviation/mean value) × 100%.

### Wavelet analysis

Wavelet analysis is a powerful tool of time-frequency localization analysis with a fixed window size and a variable shape (time width and frequency width), which has obvious advantages in multitime scale hydrological system analysis [27]. Wavelet analysis has also been recently utilized to investigate the cyclic oscillation of epidemics in a centennial period [28]. The onset cycle of HFMD has the characteristics of tendency, periodicity, and randomness. Therefore, wavelet analysis can illustrate the variance cycle in nonstationary time series of HFMD and reveal the trend and periodicity features of the system at each time scale. The generally used complex Morlet wavelet was chosen as the “mother wavelet” in this research, and MATLAB software was used to find and assess a variety of change cycles concealed in the time series of the HFMD epidemic at various time scales. Complex wavelets, compared to real wavelets, may better depict the periodicity and distribution of time series in the same time domain.

The periodic characteristics of the incidence of HFMD in Hubei were studied using wavelet analysis on three scales: day, week, and month. A wavelet coefficient contour map depicts the incidence cycle range, as well as the related incidence peak and valley variations at different time scales. A wavelet variance diagram shows the energy distribution with scale and can be used to determine the relative intensity of different scale disturbances in a time series. The major period of the time series can be represented by the scale at the peak. A wavelet coefficient’s changing process can help us understand the primary cycle trend of its time series more intuitively.

### Space-time scan statistical analysis

Spatiotemporal scan statistics were generated using SaTScan^TM^ 9.6 software (http://www.satscan.org/). The software used moving cylindrical windows to scan various times and spaces and then detected spatiotemporal high-value cluster areas of HFMD for the study period [29]. The bottom of the cylindrical window represents the scanning area, and the height of the cylindrical window is the scanning time.

Based on a discrete Poisson model, the log likelihood ratio (LLR) and relative risk (RR) were calculated for each scanning window. The most likely cluster with statistical significance was determined as the window with the highest likelihood, and other windows with lower likelihoods and statistical significance were defined as secondary clusters. The relative risk of each cluster was the ratio of the estimated risk within the cluster to that outside the cluster [29].

Affected by changes in administrative divisions such as township mergers and township renaming, there were differences in the number of townships in Hubei in different years. This study took the township division of Hubei in 2019 as the benchmark, and the data matching of other years was processed according to the administrative divisions in 2019. The spatial range of the spatiotemporal scan analysis included 1,250 villages/towns in Hubei, and the time range was set as 11 years, with a year time scale. A previous study showed that the software produces more usable and informative results depending on the choice of spatial cluster sizes [30]. We compared the results of different maximum cluster sizes (5%, 10%, 20%, 30%, 40%, and 50%) of people and chose 10% of the total population as a maximum cluster size because it performed better in detecting clusters of relatively high risk. The theoretical incidence and LLR were calculated. The p value of the LLR was calculated using a Monte Carlo randomization method. P < 0.05 was considered statistically significant.

## Results

### Epidemiological characteristics

**Fig 1:**
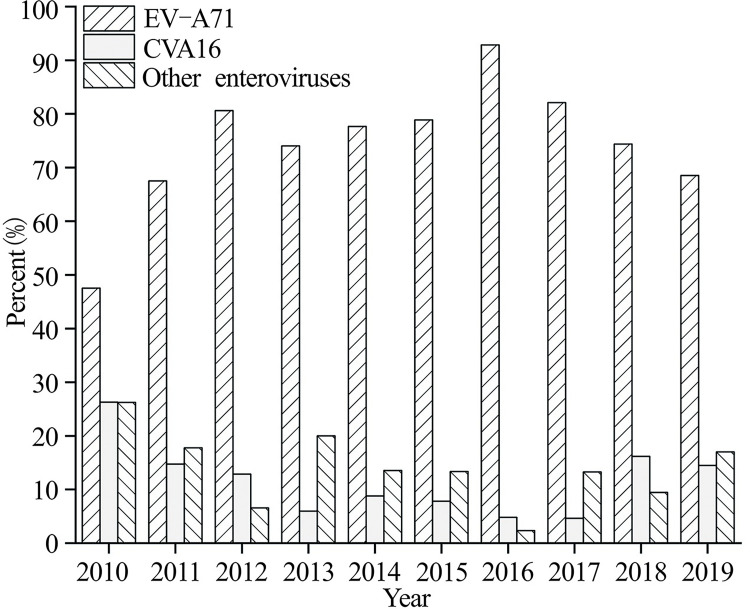
Temporal changes in the distribution of HFMD virus types in Hubei from 2010 to 2019.

HFMD is a common infectious disease caused by various types of enteroviruses. The distribution of HFMD virus types in Hubei is summarized in Fig 1, where CVA16 and EV-A71 are the two predominant causative agents, with EV-A71 being the most prevalent. Among 108,469 laboratory-confirmed cases, EV-A71, CVA16 and other enteroviruses accounted for 79.03%, 9.48% and 11.49%, respectively. The proportions of enterovirus types varied in different years. The proportion of EV-A71 was lowest in 2010 (47.52%) and highest in 2016 (92.86%). The proportions of CVA16 and other enteroviruses peaked in 2010 at 26.17% and 26.22%, and were lowest in 2016 at 4.81% and 2.34%, respectively. In general, EV-A71 was the dominant pathogen in Hubei during 2010–2019, especially in 2016. The proportion of EV-A71 decreased while the proportion of CVA16 and other enteroviruses fluctuated and increased from 2017 to 2019.

The characteristics of the pathogens consisting of EV-A71, CVA16 and other enteroviruses were similar among cities in Hubei, and the dominant strain was EV-A71 in most of the cities except for Shennongjia, which had a small population. Other enteroviruses accounted for a relatively high proportion in Shennongjia, which may be related to the small number of laboratory confirmed cases. The number of laboratory-confirmed samples of HFMD was different in each city in Hubei. For example, the number of laboratory-confirmed cases in Huangshi from 2010 to 2019 was 18,171, while the number of laboratory-confirmed cases in Shennongjia in the past 10 years was only 435.

The various enteroviruses causing HFMD in Hubei exhibited distinct spatial patterns. EV-A71 was widely distributed, with a relatively high proportion in eastern and western Hubei. CVA16 showed scattered distribution, with a relatively high proportion in southern Hubei. The higher proportion of other enteroviruses showed a transfer trend from central Hubei to eastern Hubei to western Hubei. In addition, the HFMD pathogens in Hubei ebbed and flowed. EV-A71 was dominant in all cities in Hubei from 2010 to 2016, with the highest proportion in 2016, while the proportion of CVA16 and other enteroviruses increased in some cities from 2017 to 2019, especially western Hubei.

**Fig 2:**
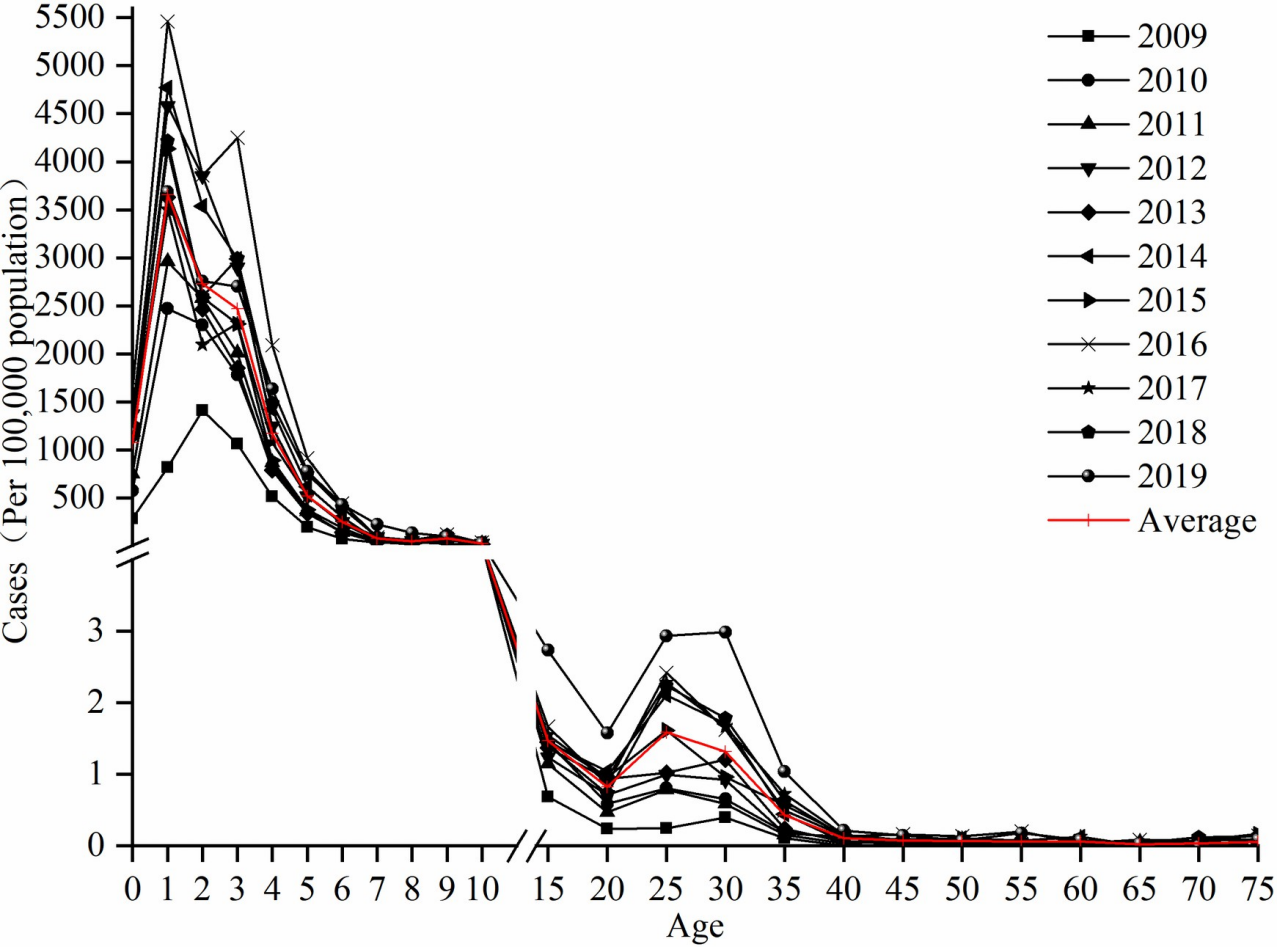
HFMD incidence by age group in Hubei from 2009 to 2019.

Fig 2 shows that the age distribution of HFMD in Hubei can be divided into two groups with 10-year intervals: the high incidence of HFMD among people under the age of 10 years mostly occurred among children aged 0–5 years, and the high incidence of HFMD among people aged over 10 years mostly occurred among people aged 25–35 years. This feature has remained stable for many years. The average incidence among children aged 0–5 years was 1,074.30/100,000, 3,657.39/100,000, 2,731.75/100,000, 2,470.10/100,000, 1,170.85/100,000, and 521.47/100,000 population, while the multiyear mean values of the incidence among those aged 25–35 years (25–30, 30–35) were 1.59/100,000 and 1.32/100,000 population.

**Fig 3:**
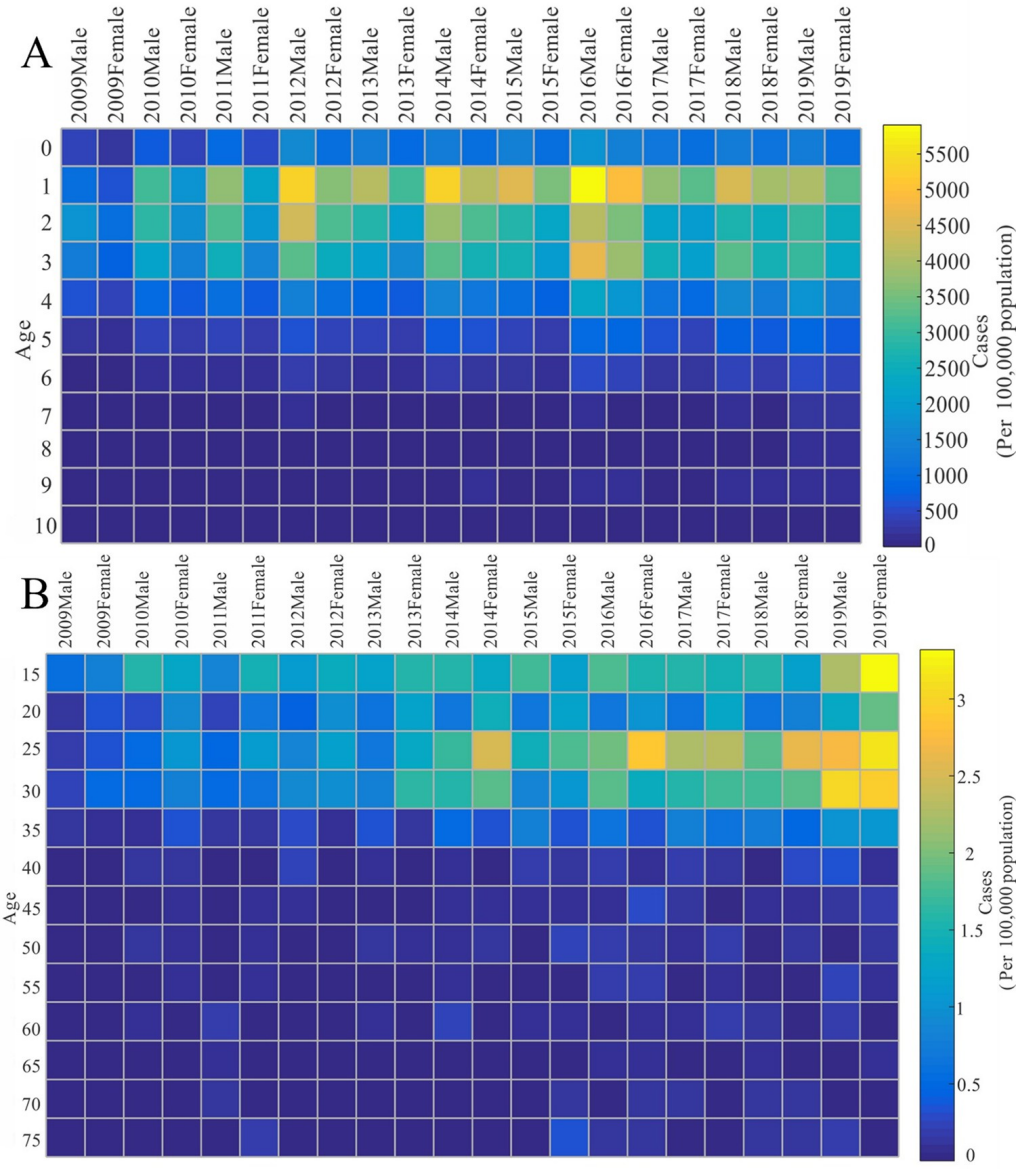
HFMD incidence by age group in Hubei from 2009 to 2019. (A) HFMD incidence in the 0–10 age group. (B) HFMD incidence in the 15–75 age group.

In the 0–10 age group (Fig 3A), the incidence among males was normally higher than that among females, especially among children aged 0–5 years. The incidence rate was highest among 1-year-olds with 4,107.09/100,000 for males and 3,125.89/100,000 for females and the sex ratio was 1.31. Compared with other years, the incidence rate among 1-year-olds peaked in 2016, with 5,905.18/100,000 for males and 4,908.50/100,000 for females, and the male-to-female ratio was 1.20.

In the age group older than 10 years (Fig 3B), the incidence was higher among 25–35 year-olds, and the incidence among females was normally higher than that among males. The incidence was 1.29/100,000 for males (25–30 years) and 1.62/100,000 for females (30–35 years). Compared with other years, the incidence rate in the 25–35 age group was highest in 2019, with 2.88/100,000 for males and 3.05/100,000 for females, and the male-to-female ratio was 0.94.

### Temporal variation characteristics

**Fig 4:**
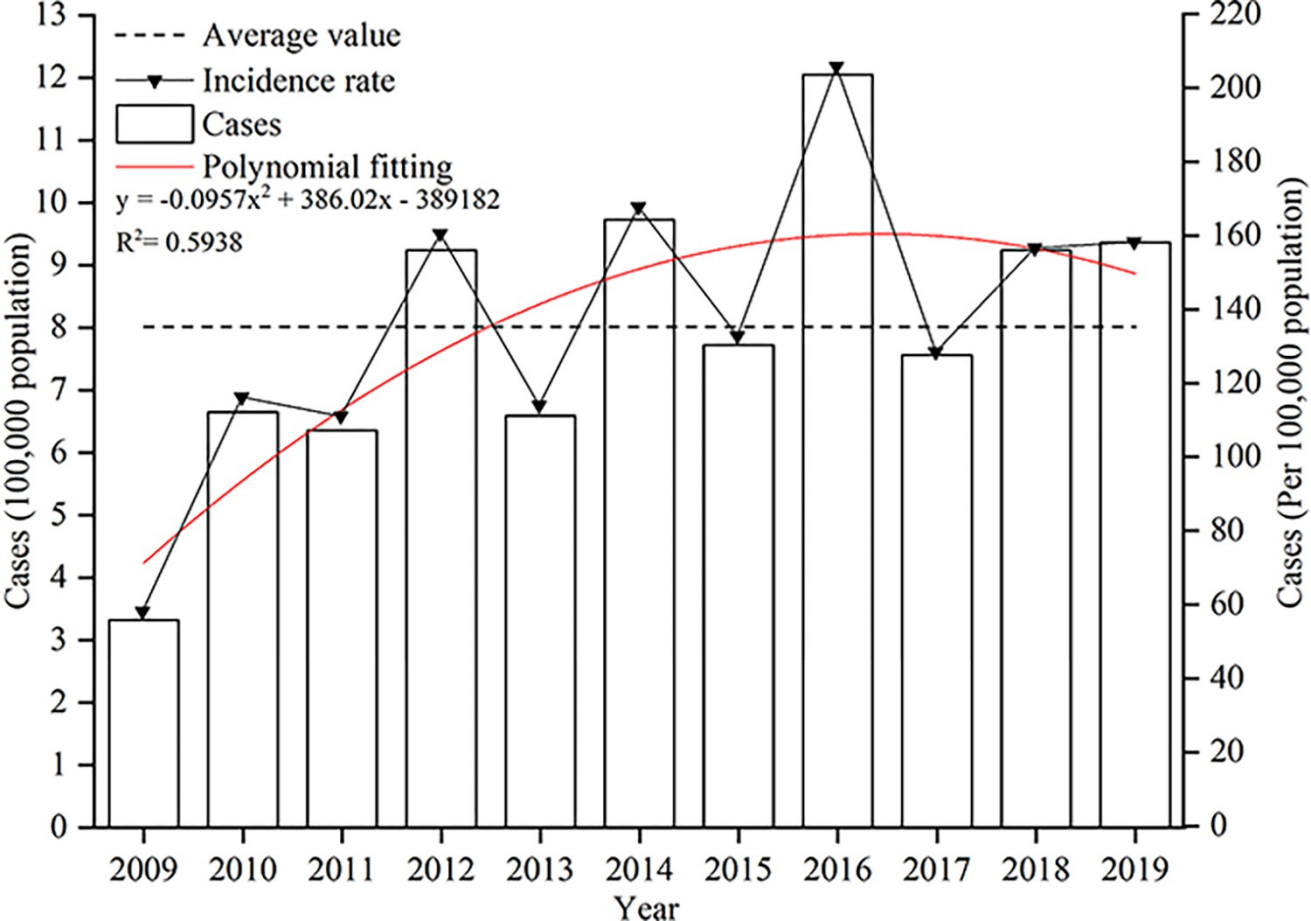
Annual variation in HFMD incidence in Hubei from 2009 to 2019.

The incidence rate of HFMD in Hubei first increased and then decreased from 2009 to 2019 (P value<0.05) (Fig 4), and the incidence in even years were greater than those in odd years. The growth rate was rapid, averaging 11.23% each year from 2009 to 2016, but it dropped from 2017 to 2019, with an average growth rate of 13.28% per year.

The number of HFMD cases increased from 33,211 in 2009 to 120,470 in 2016, and the incidence rate increased from 58.15/100,000 population to 205.88/100,000 population, with an average annual incidence of 133.30/100,000 population. The number of cases declined to 75,622 in 2017, while the incidence rate dropped to 128.50/100,000 population. Then, the number of cases increased from 75,622 in 2017 to 93,639 in 2019, and the incidence rate increased from 128.50/100,000 population to 158.25/100,000 population, for an annual incidence rate of 147.79/100,000 population. The pattern of susceptible populations may have been linked to the characteristics of high incidence in alternate years. As previously stated, HFMD is highly common among infants under the age of one year.

The HFMD epidemic had obvious seasonal characteristics. On a monthly scale, there were two peaks of HFMD in Hubei, with the larger peak observed in April-July and the smaller peak in November-December (Table 1). The seasonal indices in the first peak were all greater than 100%, with the highest index and 19.45% of the total number of cases in May. Among the secondary peaks, the largest seasonal index occurred in November, with 7.9% of the number of HFMD cases. The coefficient of variance in April-July was relatively low, showing that the seasonality of HFMD in Hubei was significant and stable.

**Table 1:** Seasonal index of HFMD incidence in Hubei.

Month	Seasonal index (%)	Standard deviation	Mean value	Variable coefficient (%)
1	44.61	1,488.80	2,968.27	0.50
2	20.00	675.49	1,330.55	0.51
3	65.57	1,813.31	4,362.91	0.42
4	172.23	3,929.01	11,459.18	0.34
5	233.34	5,830.53	15,525.45	0.38
6	199.18	5,047.89	13,252.18	0.38
7	114.21	2,946.93	7,599.09	0.39
8	45.70	1,356.66	3,040.73	0.45
9	51.44	1,095.72	3,422.55	0.32
10	70.34	2,202.31	4,680.09	0.47
11	94.36	2,610.25	6,278.09	0.42
12	89.01	2,435.24	5,922.45	0.41

**Fig 5:**
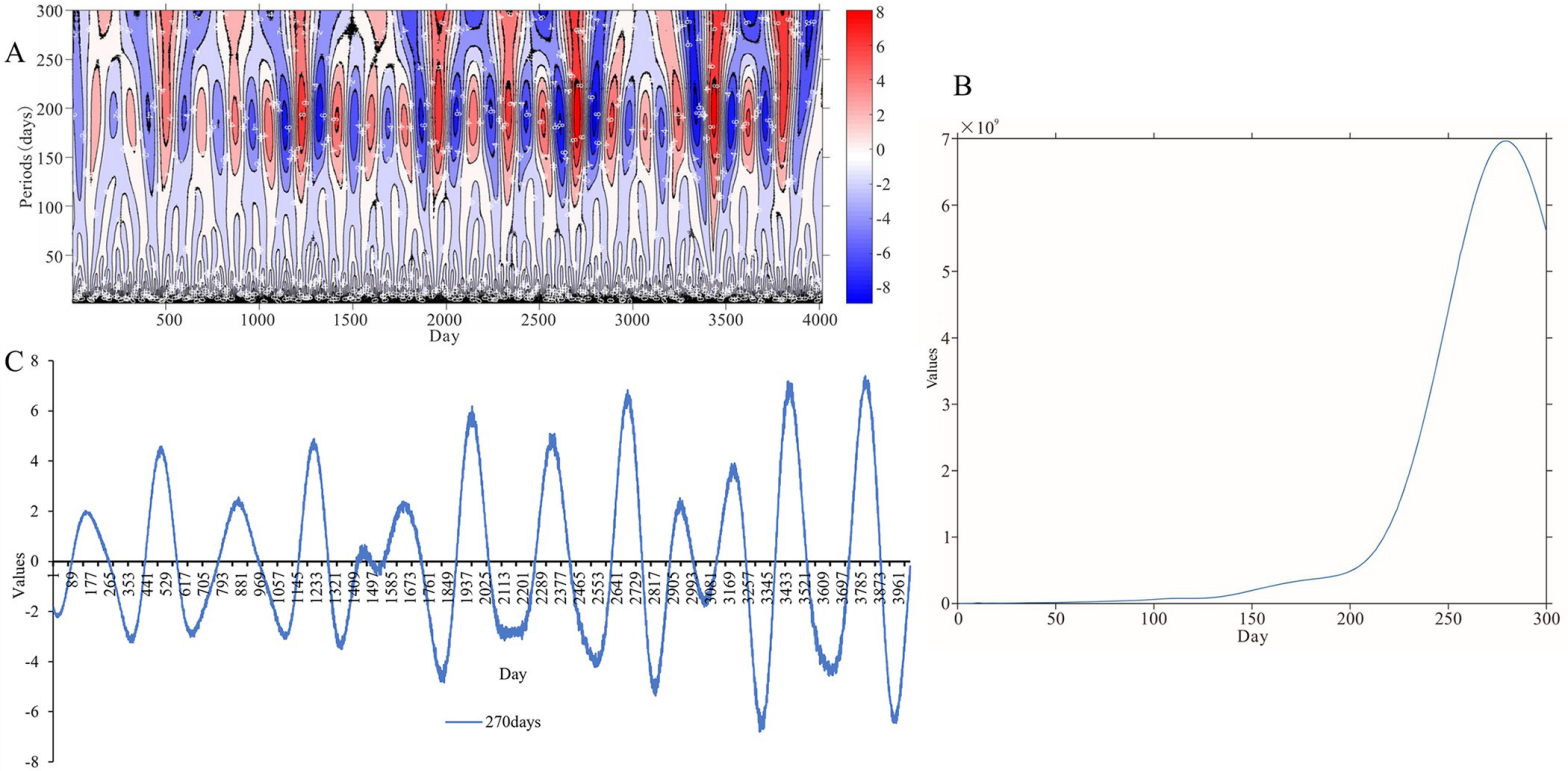
Daily time-series data and wavelet analysis of HFMD in Hubei from 2009 to 2019. (A) The real part of the wavelet coefficient of HFMD. Color is the power spectrum, strong to weak (red–blue gradient). (B) The wavelet variance of HFMD on daily scale. (C) The real part of the wavelet coefficient’s changing process on daily scale.

The wavelet analysis results are depicted in Fig 5. Taking the daily scale as an example, the center of the real part of the wavelet coefficient contour is mainly distributed in a scale range of 180–270 days (Fig 5A). The wavelet variogram has only one obvious peak, and the wavelet corresponds to 270 days, reflecting the first and only main cycle of the daily temporal variation characteristics of HFMD incidence in Hubei (Fig 5B). Under the 270-day scale, HFMD in Hubei experienced approximately 13 "light-severe" transition cycles, with an average of 150 days. The time periods with high incidence were 101–270 days, 430–580 days, 800–950 days, 1150–1300 days, 1410–1510 days, 1610–1710 days, 1900–2030 days, 2250–2400 days, 2610–2770 days, 2900–3000 days, 3100–3250 days, 3410–3550 days, and 3720–3880 days (Fig 5C).

**Fig 6:**
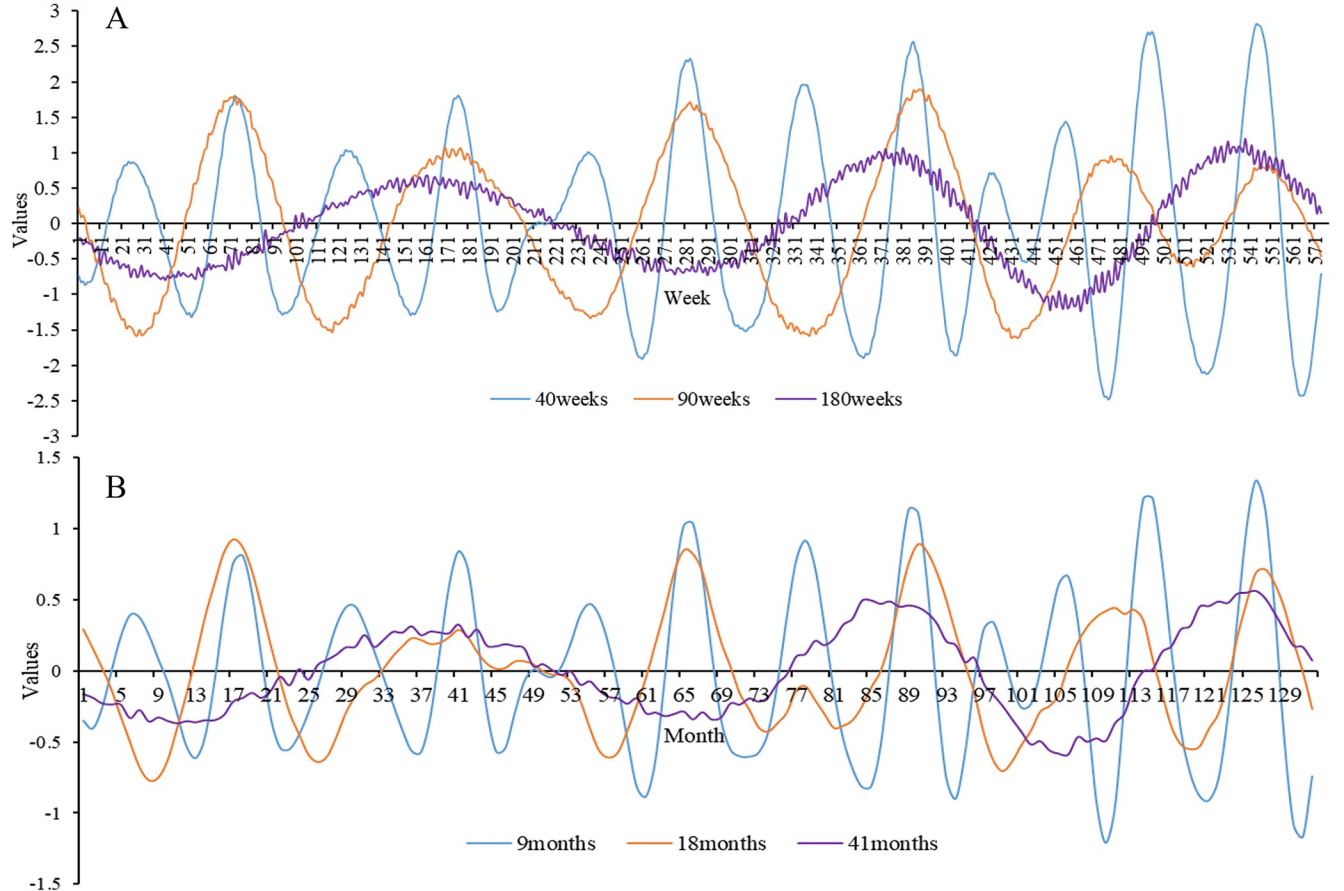
The real part of the wavelet coefficient’s changing process on weekly and monthly scales. (A) Weekly scale. (B) Monthly scale.

The periodicity of the weekly and monthly scales is briefly described in Fig 6 to prevent redundancy. 180, 90, and 40 weeks were the three primary cycles reflecting the weekly variation of HFMD in Hubei (Fig 6A), while 41, 18, and 9 months were the three main cycles indicating the monthly variation in HFMD (Fig 6B). The HFMD epidemic in Hubei exhibited a multiscale nesting phenomenon, with small-scale alterations generally nested beneath larger ones. For example, 270 days of the daily timescale were roughly equivalent to 40 weeks of the weekly timescale and 9 months of monthly timescale.

**Fig 7:**
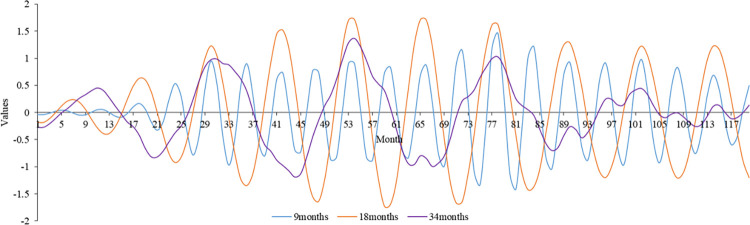
The real part of the wavelet coefficient’s changing process of cases caused by EV-A71 on a monthly scale.

We used wavelet analysis to explore the monthly epidemic cycle of HFMD cases caused by EV-A71 due to limited available data. Fig 7 shows that 34, 18, and 9 months were the three main cycles indicating the monthly variation in HFMD cases caused by EV-A71. The minimum periodicity of HFMD cases caused by EV-A71 was 9 months, which was consistent with the periodicity of HFMD cases. This demonstrates that EV-A71 vaccination could have a role in reducing the prevalence of HFMD cases.

### Temporal and spatial distribution

**Fig 8:**
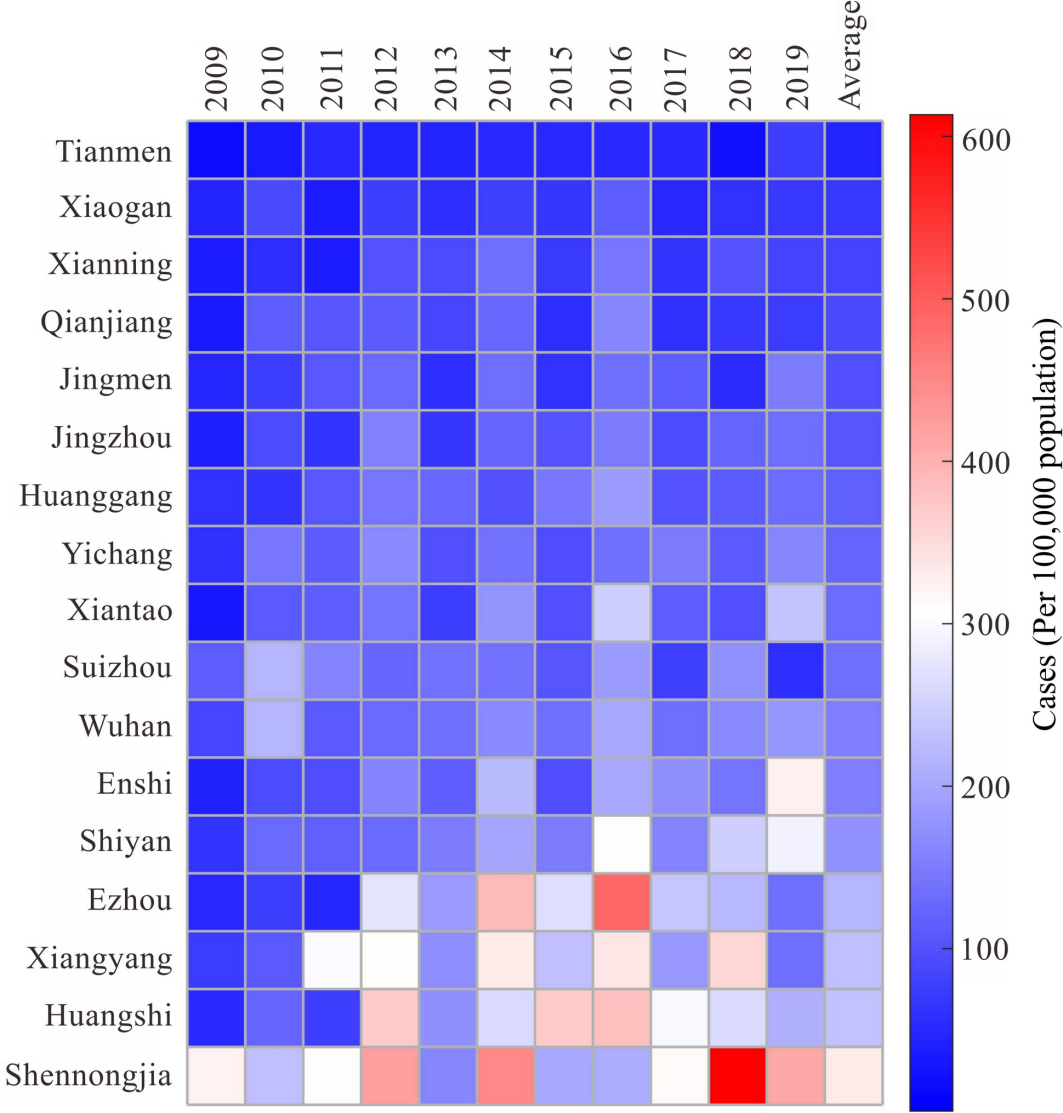
Comparison of the annual incidence rates of 17 cities in Hubei from 2009 to 2019.

At the city level, the incidence rate exceeded the annual average (137/100,000) in seven cities in Hubei, in descending order as follows: Shennongjia, Huangshi, Xiangyang, Ezhou, Shiyan, Enshi, and Wuhan (Fig 8). Among them, Wuhan had a relatively low incidence rate because of a higher population density (12,326,500 population in 2019), while Shennongjia (only 76,100 population in 2019), Ezhou (1,059,700 population in 2019) and Huangshi (2,471,700 population in 2019) had relatively higher incidence rates due to lower population density. Notably, the distribution of these seven cities was generally consistent with the "A-shaped urban skeleton" [31]. The A-shaped urban skeleton refers to the regional development pole-axis system with Wuhan as the "head", Yichang and Xiangyang as the "waist", and Shiyan and Enshi as the "feet".

The classification of administrative divisions in China is different from that in other countries. Although subdistricts (*Jiedao* in Chinese, which means some areas made of blocks downtown), towns and villages are all part of the township scale, there are considerable disparities in the degree of urbanization in China, so we compared the HFMD incidence of the above three. Referring to the average annual incidence rate at the provincial level (137/100,000) and the country level (117/100,000) [32], the HFMD incidence was classified into 5 levels (0-61/100,000, 61-124/100,000, 124-214/100,000, 214-375/100,000, and 375–1,768/100,000) by combining the natural break classes in ArcGIS (see Table 2).

**Table 2:** Proportion variation of HFMD incidence in towns in Hubei from 2009 to 2019.

	(0-61/100,000)			(61-124/100,000)			(124-214/100,000)			(214-375/100,000)			375–1,768/100,000)		
	Village	Town	Subdistrict	Village	Town	Subdistrict	Village	Town	Subdistrict	Village	Town	Subdistrict	Village	Town	Subdistrict
Year	(%)	(%)	(%)	(%)	(%)	(%)	(%)	(%)	(%)	(%)	(%)	(%)	(%)	(%)	(%)
2009	19.36	53.12	17.04	0.4	4	3.44	0.08	0.56	1.52	0	0.16	0.24	0	0	0.08
2010	17.68	47.92	9.92	1.6	7.2	6.24	0.32	2	3.36	0.08	0.56	1.92	0	0.16	0.88
2011	18.56	46.96	12.48	0.64	6.24	4.24	0.56	3.36	2.8	0	0.96	2.48	0.08	0.32	0.32
2012	16.4	37.52	9.28	2.24	12.64	5.6	1.12	5.2	3.52	0	1.84	3.12	0.08	0.64	0.8
2013	18.32	46.88	10.32	1.2	7.28	6.48	0.24	2.48	3.04	0	1.04	1.84	0.08	0.16	0.64
2014	16.72	38.96	8.56	2.48	11.36	5.28	0.48	4.72	3.68	0.08	2.16	3.52	0.08	0.64	1.28
2015	18.08	44.48	9.12	1.44	8.56	5.76	0.16	2.8	4.16	0	1.44	2.48	0.16	0.56	0.8
2016	15.84	35.52	6.16	3.28	12.24	5.52	0.48	6.32	4.24	0.16	2.48	3.92	0.08	1.28	2.48
2017	18.08	44.96	9.76	1.28	8.72	5.52	0.4	2.64	3.04	0	1.2	2.56	0.08	0.32	1.44
2018	18	43.44	8.64	1.44	8.8	5.28	0.24	2.88	3.84	0.08	1.84	2.8	0.08	0.88	1.76
2019	17.36	45.36	10	1.6	7.6	4.24	0.56	2.24	3.12	0.16	1.84	2.64	0.16	0.8	2.32

At the town level, the areas with higher incidence rates expanded, whereas the areas with lower incidence rates shrank between 2009 and 2019. Areas with an incidence of 0-61/100,000 and 61-124/100,000 were the most common, together accounting for 97.36% of the incidence in 2009 and falling to 86.16% in 2019. The regions with incidence rates of 124-214/100,00, 214-375/100,000, and 375–1,768/100,000 together accounted for only 2.64% of the incidence in 2009, but by 2019 these regions accounted for 13.84% of the incidence. These results indicate that there was a spreading trend of HFMD prevalence in Hubei.

The variation in HFMD incidence in the three administrative divisions was in descending order: subdistrict > town > village. The number of towns with incidence rates of 0-61/100,000 and 61-124/100,000 showed the most variation. Among them, the number of areas with an incidence of 0-61/100,000 declined by 7.76%, while the number of areas with an incidence rate of 61-124/100,000 increased by 3.60%. The greatest changes in the number of subdistricts with incidence rates were observed for areas with incidence rates of 124-214/100,000, 214-375/100,000, and 375–1,768/100,000. The three types of areas increased by 1.60%, 2.40% and 2.24%, respectively. The variation in the number of villages was smaller in all five classification levels, among which, areas with incidence of 0-61/100,000 showed the largest change, with a 2% decrease over 11 years.

The incidence rates in even years were higher than those in odd years. Taking 2016, during which the incidence rate was the highest, as an example, the number of areas with an incidence of 0-61/100,000 varied the most. The number of villages, towns, and subdistricts where HFMD was prevalent decreased by 3.52%, 17.60% and 10.88% respectively. After 2016, the number of areas with an incidence of 0-61/100,000 increased, which also indicated that the severity of HFMD epidemics in Hubei showed a downward trend after 2016.

HFMD clusters were more evident and had a wider range aggregation in western and central Hubei, particularly in the main urban areas and surrounding counties, but HFMD clusters in eastern Hubei were more dispersed and mainly located in the centers of the main urban areas. The high-incidence HFMD zones displayed a trend of “east‒west” fluctuation. Detailed information about the spatiotemporal scan results can be found in Table 3.

**Table 3:** Spatiotemporal clusters of HFMD in Hubei from 2009 to 2019.

Class of cluster	Cluster center/Radius	Time	N	Observed value	Expected value	RR value	LLR value	P value
2	(31.60 N, 113.91 E)/18.90 km	2010/1/1-2010/12/31	10	1,897	688.65	2.76	714.75	0.00
2	(30.52 N, 112.75 E)/0.00 km	2010/1/1-2010/12/31	1	138	54.66	2.53	44.47	0.00
2	(31.01 N, 113.74 E)/0.00 km	2010/1/1-2010/12/31	1	171	51.84	3.30	84.94	0.00
2	(30.56 N, 111.94 E)/65.16 km	2012/1/1-2012/12/31	108	11,308	7,144.85	1.59	1,039.12	0.00
2	(31.12 N, 113.87 E)/0.00 km	2012/1/1-2012/12/31	1	231	61.91	3.73	135.08	0.00
2	(31.20 N, 113.69 E)/0.00 km	2012/1/1-2012/12/31	1	293	35.71	8.21	359.43	0.00
2	(30.67 N, 114.26 E)/19.78 km	2016/1/1-2016/12/31	94	11,489	7,373.78	1.57	990.00	0.00
2	(30.36 N, 114.92 E)/28.04 km	2016/1/1-2016/12/31	51	11,016	2,891.53	3.85	6,650.18	0.00
2	(31.22 N, 115.01 E)/31.99 km	2016/1/1-2016/12/31	17	2,701	1,151.01	2.35	755.37	0.00
2	(30.33 N, 113.07 E)/21.65 km	2016/1/1-2016/12/31	13	1,554	1,019.36	1.53	120.79	0.00
2	(29.84 N, 114.34 E)/17.81 km	2016/1/1-2016/12/31	11	2,050	742.76	2.76	774.99	0.00
2	(29.43 N, 113.99 E)/15.98 km	2016/1/1-2016/12/31	5	460	309.69	1.49	31.70	0.00
2	(30.93 N, 113.56 E)/0.00 km	2016/1/1-2016/12/31	1	252	166.58	1.51	18.90	0.00
2	(31.25 N, 113.99 E)/0.00 km	2016/1/1-2016/12/31	1	483	158.66	3.05	213.43	0.00
2	(30.51 N, 112.92 E)/0.00 km	2016/1/1-2016/12/31	1	156	80.24	1.94	27.95	0.00
2	(30.73 N, 112.79 E)/0.00 km	2017/1/1-2019/12/31	1	173	61.68	2.81	67.12	0.00
**1**	**(32.09 N, 111.44 E)/90.51 km**	**2018/1/1-2018/12/31**	**121**	**20,042**	**7,564.83**	**2.69**	**7,145.36**	**0.00**
2	(31.66 N, 113.48 E)/16.62 km	2018/1/1-2018/12/31	6	1,568	737.71	2.13	352.41	0.00
2	(30.49 N, 114.06 E)/8.42 km	2018/1/1-2018/12/31	3	2,195	246.45	8.93	2,853.69	0.00
2	(29.72 N, 112.44 E)/0.00 km	2018/1/1-2018/12/31	1	239	13.70	17.45	458.04	0.00
2	(30.18 N, 109.25 E)/36.91 km	2019/1/1-2019/12/31	20	5,760	1,310.46	4.42	4,090.45	0.00
2	(30.33 N, 113.43 E)/7.00 km	2019/1/1-2019/12/31	3	1,291	401.99	3.21	617.73	0.00
2	(31.16 N, 112.68 E)/7.56 km	2019/1/1-2019/12/31	2	424	87.26	4.86	333.61	0.00

There were 23 spatiotemporal clusters found, with the most likely cluster being discovered in Xiangyang in 2018 and the remainder being secondary clusters observed in various years. In 2010, secondary clusters were found in eastern Suizhou city (including 10 towns) as well as one town in Xiaogan and another in Qianjiang. In 2012, a secondary cluster that included 108 towns was found at the juncture of Yichang, Jingmen, and Jingzhou cities. In 2016, the secondary cluster was mainly distributed in the urban downtown area in central and eastern Hubei, such as Wuhan, Ezhou, Huangshi, and Huanggang. The largest cluster was located in Wuhan and included 94 towns. In 2017, only one town in Tianmen was observed to belong to a cluster. In 2018, the most likely cluster was detected in Xiangyang, Shiyan, and Shennongjia, with a total of 121 towns, the secondary clusters were observed in downtown Suizhou, Wuhan, and Jingzhou. In 2019, a secondary cluster occurred in Enshi, Jingmen, and Xiantao, and the largest cluster consisting of 20 towns was found in Enshi. In addition, this study found that the spatial and temporal clusters were coincident with the geographic location of railways and rivers in Hubei.

## Discussion

The predominant pathogen of HFMD in Hubei was EV-A71, which was consistent with that of Jingzhou city [33], and Xiangyang city [34]. Our study showed that EV-A71 accounted for the highest proportion in all cities in Hubei in 2016. This may account for the high incidence of HFMD in Hubei in that year. Compared with those infected with CVA16, children infected with EV-A71 tended to have more severe HFMD symptoms. It is suggested that areas with a high proportion of EV-A7 properly strengthen the publicity of EV-A71 vaccination and encourage children to get vaccinated to decrease incidence of HFMD. In addition, the proportion of CVA16 and other enteroviruses showed a fluctuating increase from 2017 to 2019, especially in western Hubei. The proportion of CVA16 in Beijing has increased since 2018 [35]. A decrease in EV-A71 HFMD cases was observed in Xiangyang city at the beginning of 2017, 2 to 3 months after vaccination, along with an increase in cases of CVA2 and CVA5 [36]. Our study showed that the proportion of EV-A71 decreased after 2016, while the proportion of CVA16 increased in Hubei. This indicated that there was a changing trend of dominant strains in HFMD, including the decline in the proportion of EV-A71 and the increase in the proportion of Coxsackie virus. Notably, the available HFMD vaccines only had a good protective effect against EV-A71 and could not form crossover protection against other enteroviruses. In this regard, it is also essential to help children develop good health habits, pay attention to the hygienic environment of kindergartens and nurseries, and enhance the morning check system and other preventive measures in western Hubei.

The population (age and sex) characteristics of the HFMD epidemic in Hubei were similar to those in Shandong [8], Yunnan [37] and Guangdong provinces [38] in China and were also consistent with those in a study in Singapore [6]. The prevalence of HFMD among male children under 10 years is generally higher than that among female children. A likely explanation is that boys are more active and exposed to the HFMD virus environment than girls. In contrast to existing studies, we found a significantly higher incidence among females than males aged 25–35 years, which might be partly because females in this age group are more likely to be responsible for the care of children under 5 years of age, and close contact with children increases the risk of HFMD infection. This finding might also be related to the delayed age of childbearing among women of childbearing age and the full implementation of the two-child policy in 2013. Therefore, children aged 0–5 years should be a focus for priority attention, and young mothers aged 25–30 years should receive more training and better information about HFMD to protect themselves when caring for children with HFMD.

HFMD incidence in Hubei witnessed two peaks in April-July and November-December, which corresponded to the epidemic features in Yunnan province [37] and Hangzhou city [39]. Previous studies have shown that HFMD in southern cities of China has two peaks and that northern cities have one peak [40]. This may be due to meteorological factors associated with the incidence of HFMD. The environment in which enteroviruses reproduce is closely related to meteorological factors. Many studies have illustrated that temperature, humidity, and precipitation significantly affect the incidence of HFMD [23, 41]. In Hubei, the HFMD epidemic peaked in April-July and December-November. During these two periods, families, kindergartens, and communities should focus on HFMD prevention and the cleaning and disinfection of children’s play areas.

HFMD in Hubei had a 270-day periodicity, according to this study, and there was multiscale nesting between distinct scales. The periodicity of HFMD cases caused by EV-A71 infection was 9 months. Wavelet analysis was introduced to the field of atmospheric sciences and hydrology in the 1990s [42, 43]. In recent years, several researchers have begun to use wavelet analysis to study diseases. Wavelet analysis, for example, has been used to investigate the periodicity of the main enterovirus strains of HFMD [44]. However, limited research has been conducted on the cyclical elements of HFMD incidence rates. Xia et al. showed that the time series of HFMD cases in Northwest China has an obvious annual cycle and semiannual cycle [45], which is different from the result of the 270-day periodicity in Hubei. This may indicate that the periodicity of HFMD incidence varies in different regions. As mentioned above, HFMD in southern China has two peaks, while HFMD in northern China has only one peak. Research on the cyclical patterns of the HFMD incidence rate could provide a foundation for future prediction and early warning systems.

We found that the range of areas with high prevalence rates of HFMD in Hubei has expanded, whereas the range of areas with low prevalence rates has shrunk. This conclusion is mostly in line with Gong’s findings [24]. However, there are also differences. For example, 2016 was a turning point. The range of areas with lower HFMD incidence rates expanded after 2016, and the prevalence of HFMD in Hubei slowed slightly, probably related to the implementation of the EV-A71 vaccine in 2016, with some children receiving the HFMD vaccine. As previously mentioned, the epidemic regularity of HFMD cases caused by EV-A71 was consistent with the periodicity of HFMD. Despite the fact that Xiangyang was a pilot city for HFMD vaccination, the results of the spatiotemporal scan showed that the disease was more prevalent in Xiangyang than elsewhere in 2018. It is necessary to continue to promote HFMD vaccination while paying more attention to geographic environments that may result in areas with high disease incidence rates.

The results of the spatiotemporal scan revealed that clusters were primarily observed in downtown urban areas and surrounding counties or towns, especially in areas that crossed various cities. These findings were similar to those of Wang’s research [40]. In metropolitan regions, high population density, population mobility, and developed transportation facilitate the spread of HFMD, whereas in less developed areas, limited health care makes HFMD control difficult. These observations suggest that the preventative and control measures utilized in developed areas might need to differ from those used in less developed areas, and communities need to work closely together. The numbers of cases in economically developed areas, such as along the “A-shaped urban skeleton” are higher because of a dense population, while the incidence rates in less developed areas are higher because of insufficient health resources and lack of health awareness. Therefore, the former, such as Wuhan, Xiangyang, and Yichang, need to pay more attention to areas with a high concentration of young children, especially kindergarten children, while the latter, such as Ezhou, Suizhou, Enshi and Shennongjia, should increase local medical resources and awareness of HFMD prevention and control. Vaccination against HFMD should also be promoted in areas where HFMD is widespread. This study also found that areas with high HFMD incidence rates were coincident with the distribution of rivers and railways, implying that the association between the geographic environment surrounding rivers and the prevalence of HFMD should be examined further. In addition, studies are needed to determine whether railroads are a proxy for people spreading HFMD as they travel among communities or a proxy for environmental conditions adjacent to railroad lines.

There are some limitations to this study. First, regarding the spatiotemporal scale, restricted by data availability, when the spatial scale was based on townships, the corresponding time scale was years rather than months. If HFMD data with months as the time dimension and towns as the spatial resolution could be obtained, spatiotemporal characteristics might be better illustrated. Second, the regional differences in wavelet analysis should be investigated. There may be geographical differences in the cycle of HFMD incidence in different regions as well as the cycle characteristics of epidemic strains. Furthermore, the relationships between meteorological factors and socioeconomic factors need to be further explored.

## Conclusions

HFMD is a common infectious disease that mainly affects children under the age of 5 years and presents a substantial threat to children’s health. EV-A71 and CVA16 were the two main pathogens of HFMD in Hubei, among which EV-A71 was the dominant pathogen, especially in 2016. It is necessary to strengthen the publicity of the EV-A71 vaccine in areas with a high proportion of EV-A71. Children under the age of 5, as well as people between the ages of 25 to 35, are particularly susceptible. The number/incidence of HFMD cases in Hubei increased substantially, followed by a moderate decline, presenting two peaks per year. Early prevention and control measures need to be prioritized before April-July and November-December in Hubei. Studies of other places where HFMD is common are needed to determine their peak periods. HFMD in Hubei also exhibited the characteristics of a 270-day cycle, which was similar to the period of HFMD cases caused by EV-A71 infection (9 months). The inactivated EV-A71 vaccine may be of great importance to control the HFMD epidemic. The HFMD epidemic showed an upward trend followed by a downward trend. Spatiotemporal clusters were consistent with " A-shaped urban skeleton" cities as well as the geographic location of railways and rivers in Hubei. More attention should be given to the incidence of HFMD in " A-shaped urban skeleton" cities (Xiangyang, Wuhan, Ezhou, Huangshi, and Enshi) and in other city systems. These findings could be helpful for the prevention and control of HFMD in certain time periods and key areas in Hubei and the expansion of these types of studies to other regions would help reduce HFMD incidence rates not only in China but also in other parts of the world where it is common.

## Supporting information

S1 Table The distribution of HFMD virus types in 17 cities in Hubei from 2010 to 2019. (XLSX)

S2 Table Spatiotemporal clusters of HFMD in Hubei from 2009 to 2019. (ZIP)

S3 Table The annual incidence of HFMD in 17 cities in Hubei from 2009 to 2019. (XLSX)

S1 Fig Age distribution of HFMD incidence in Hubei. (OPJ)

S2 Fig Temporal characteristics of HFMD cases in Hubei at different time scales (year, month, week, day). (OPJ)
